# Clinical Grade Treg: GMP Isolation, Improvement of Purity by CD127^pos^ Depletion, Treg Expansion, and Treg Cryopreservation

**DOI:** 10.1371/journal.pone.0003161

**Published:** 2008-09-08

**Authors:** Jorieke H. Peters, Frank W. Preijers, Rob Woestenenk, Luuk B. Hilbrands, Hans J. P. M. Koenen, Irma Joosten

**Affiliations:** 1 Department of Bloodtransfusion and Transplantation Immunology, Radboud University Nijmegen Medical Centre, Nijmegen, the Netherlands; 2 Central Hematology Laboratory, Radboud University Nijmegen Medical Centre, Nijmegen, the Netherlands; 3 Department of Nephrology, Radboud University Nijmegen Medical Centre, Nijmegen, the Netherlands; New York University School of Medicine, United States of America

## Abstract

**Background:**

Treg based immunotherapy is of great interest to facilitate tolerance in autoimmunity and transplantation. For clinical trials, it is essential to have a clinical grade Treg isolation protocol in accordance with Good Manufacturing Practice (GMP) guidelines. To obtain sufficient Treg for immunotherapy, subsequent *ex vivo* expansion might be needed.

**Methodology/Principal Findings:**

Treg were isolated from leukapheresis products by CliniMACS based GMP isolation strategies, using anti-CD25, anti-CD8 and anti-CD19 coated microbeads. CliniMACS isolation procedures led to 40–60% pure CD4^pos^CD25^high^FoxP3^pos^ Treg populations that were anergic and had moderate suppressive activity. Such CliniMACS isolated Treg populations could be expanded with maintenance of suppressive function. Alloantigen stimulated expansion caused an enrichment of alloantigen-specific Treg. Depletion of unwanted CD19^pos^ cells during CliniMACS Treg isolation proved necessary to prevent B-cell outgrowth during expansion. CD4^pos^CD127^pos^ conventional T cells were the major contaminating cell type in CliniMACS isolated Treg populations. Depletion of CD127^pos^ cells improved the purity of CD4^pos^CD25^high^FoxP3^pos^ Treg in CliniMACS isolated cell populations to approximately 90%. Expanded CD127^neg^ CliniMACS isolated Treg populations showed very potent suppressive capacity and high FoxP3 expression. Furthermore, our data show that cryopreservation of CliniMACS isolated Treg is feasible, but that activation after thawing is necessary to restore suppressive potential.

**Conclusions/Significance:**

The feasibility of Treg based therapy is widely accepted, provided that tailor-made clinical grade procedures for isolation and *ex vivo* cell handling are available. We here provide further support for this approach by showing that a high Treg purity can be reached, and that isolated cells can be cryopreserved and expanded successfully.

## Introduction

Regulatory T cells (Treg) play a critical role in various immunological processes, particularly in maintaining homeostasis and self-tolerance. Immunotherapy based on Treg infusion is therefore a potential treatment for many immune disorders. This approach has been proven successful in animal models of stem cell transplantation [1]–[7], solid organ transplantation [8], [9] and auto-immunity [6], [10]–[13]. Therapeutic application in humans requires large numbers of Treg, that have to be isolated and, if necessary, expanded using clinical grade (Good Manufacturing Practice, GMP) protocols. The CliniMACS system provides a relatively versatile method for GMP cell isolation and is able to manage large quantities of cells, for example from leukapheresis material.

Other authors have recently shown the feasibility of CliniMACS for CD4^pos^CD25^high^ Treg enrichment, but failed to achieve Treg purity of more than 40–60% [14], [15]. For stem cell transplantation recipients, a suboptimal Treg purity is unlikely to be harmful. Currently, many stem cell transplantation recipients are treated with donor lymphocyte infusions to enhance graft-versus-leukemia/tumor responses. The main adverse effect of this treatment is graft-versus-host-disease (GVHD). The aim of Treg immunotherapy in this patient group is to reduce these graft-versus-host responses. Co-infusion of both non-regulatory T cells to ensure graft-versus-leukemia/tumor responses and Treg to prevent excessive GVHD is therefore a logical approach in this setting. However, in other patient groups, such as solid organ graft recipients and patients with auto-immune diseases, high purity of Treg for immunotherapy is crucial. Infusion of non-regulatory cells into patients that already suffer from unwanted immunological activity should be prevented, as these cells can potentially intensify the disease process. The main aggressive cell types in immune responses are cytotoxic CD8^pos^ T cells and CD4^pos^ conventional T cells, and contamination of CliniMACS isolated Treg populations with these cells should be avoided for immunotherapy in patients other than stem cell recipients. Likewise, contamination of the isolate with B cells could lead to the infusion of activated B cells with potential adverse immunological consequences.

Although CliniMACS based Treg isolation from leukapheresis products will yield large numbers of Treg, *ex vivo* expansion of these cells prior to infusion might still be necessary to obtain sufficient numbers of Treg for effective immunotherapy. Treg can be expanded efficiently using polyclonal stimulation [16]–[19]. However, antigen-specific Treg have been shown to be far more efficient than polyclonal Treg in animal models [3], [5]–[7], [12], [13]. In allogeneic transplantation, the target antigens are known (mainly foreign HLA) and human alloantigen-specific Treg can be obtained by expansion using stimulation with alloantigen [20], [21].

For practical reasons, it would be convenient or even necessary to perform the leukapheresis procedure weeks/months before the transplantation or treatment and store the Treg until they are needed. Currently, information on cryopreservation of Treg is lacking.

In the current study, we confirm that GMP Treg isolation using standard CliniMACS procedures results in moderately pure Treg populations. Here, we show that purity of CliniMACS isolated Treg can be improved by depletion of CD127^pos^ cells. In addition, we show that CliniMACS isolated Treg can be expanded with maintenance of suppressive function using polyclonal or alloantigen stimuli in the presence of IL-2. Depletion of CD19^pos^ B cells during CliniMACS Treg isolation appears to be a prerequisite to prevent B cell outgrowth during expansion. Furthermore, we show that cryopreservation of Treg is feasible, but that the cells require activation to restore suppressive potential.

## Results

### Isolation of Treg using CliniMACS yields 40–60% pure CD4^pos^CD25^high^ Treg populations with suppressive activity

Treg immunotherapy will demand very high numbers of highly pure Treg populations isolated in a GMP manner. To obtain large numbers of Treg with optimal purity, we performed CD4^pos^CD25^high^ T cell isolation from healthy donor leukapheresis products using the GMP CliniMACS system. The most straight-forward strategy to enrich for Treg would be a one step positive selection of CD25^pos^ cells out of leukapheresis products. However, this strategy would unintentionally result in contaminating (activated) cytotoxic CD8^pos^ T cells in the isolated Treg populations. Therefore, our initial CliniMACS isolation strategy consisted of two steps: first a CD8 depletion step to exclude CD8^pos^ effector T cells from the isolated cell populations, followed by a CD25 enrichment step to enrich for CD4^pos^CD25^high^ Treg (referred to as CliniMACS^8/25^). For comparison, a laboratory scale MiniMACS based Treg isolation protocol was also performed (referred to as MiniMACS).

**Figure 1:**
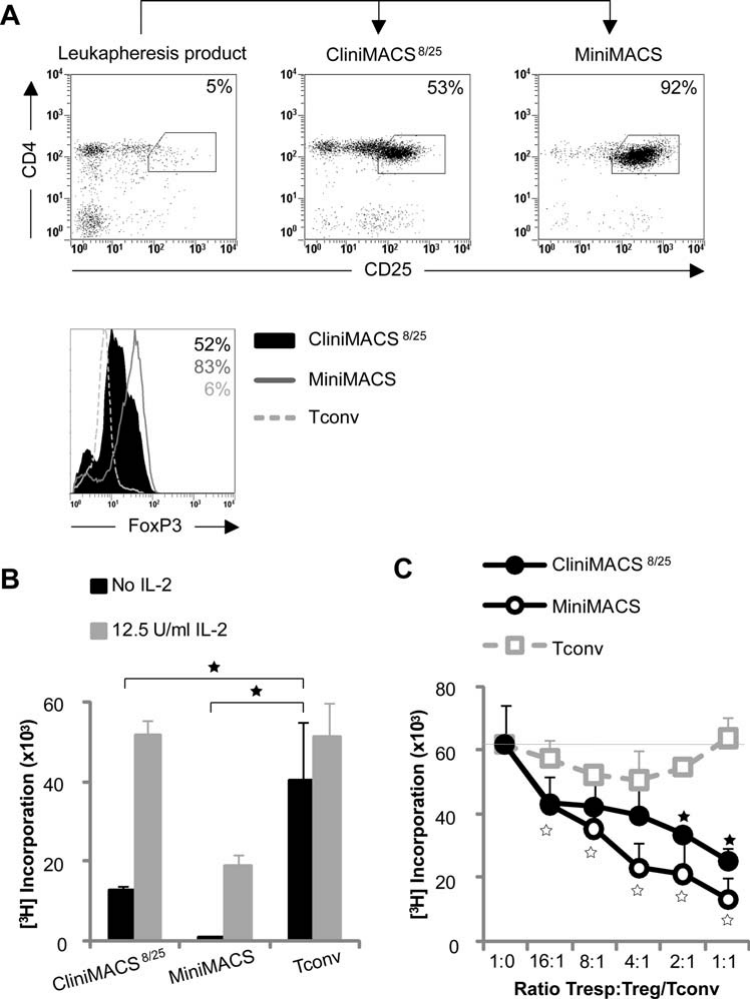
Phenotypic and functional characteristics of freshly CliniMACS isolated Treg. Treg were isolated from leukapheresis products in GMP CliniMACS-based or non-GMP MiniMACS-based isolation procedures. Data from a typical isolation are shown (N = 3). (A) Cell surface expression of CD4 and CD25 and intracellular expression of FoxP3. (B) Proliferative capacity upon stimulation with allogeneic PBMC in the absence or presence of exogenous IL-2, measured at day 5 of culture. (C) Suppressive capacity in co-cultures of autologous naïve CD4^pos^CD25^neg^ Tresp stimulated with allogeneic PBMC. Significant differences are indicated by asterisks. In figure C, open and filled asterisks refer to Tconv (used as control for the addition of cells) versus MiniMACS or CliniMACS^8/25^, respectively.

Leukapheresis products contained on average 3% CD4^pos^CD25^high^ regulatory T cells (Figure 1 and Table 1). Less than 0.1% CD8^pos^ cells were present in the isolated CliniMACS^8/25^ populations. While 80% of CliniMACS^8/25^ cells was CD4^pos^CD25^pos^, only 40–60% was CD4^pos^CD25^high^ (Figure 1A and Table 1). MiniMACS based Treg isolation from the same leukapheresis product yielded a higher Treg purity with 90% CD4^pos^CD25^high^ cells. CliniMACS^8/25^ populations expressed lower levels of FoxP3 as compared to MiniMACS isolated Treg populations (Figure 1A).

**Table 1:** Enrichment of CD4^pos^CD25^high^ Treg by CliniMACS isolation.

	Number of WBC×10^8^ (SD)	Percentage of cells with indicated phenotype (SD)				
		CD4^pos^	CD4^pos^	CD4^pos^	CD8^pos^	CD20^pos^
		CD25^high^	CD25^pos^	T cell	T cell	B cell
**Leukapheresis** *	27.2 (1.9)	3 (3)	8 (8)	60 (1)	26 (7)	16 (15)
**CliniMACS^8/25^**	1.5 (0.0)	45 (19)	78 (0)	95 (1)	0 (0)	2 (1)
**CliniMACS^8/19/25^**	1.3 (0.1)	46 (13)	81 (5)	97 (1)	0 (0)	0 (0)
**MiniMACS**	-	88 (2)	90 (1)	98 (0)	1 (1)	1 (1)

N = 3.

* Partial leukapheresis products were used.

A hallmark of CD4^pos^CD25^high^ Treg is their anergic phenotype, defined as low proliferative capacity upon T cell receptor stimulation alone as compared to Tconv cells, which can be restored by the addition of exogenous IL-2. Both CliniMACS^8/25^ and MiniMACS populations were anergic (Figure 1B).

Suppressive capacity of Treg populations was determined by titration of Treg into co-cultures of autologous CD4^pos^CD25^neg^ responder T cells stimulated with allogeneic stimulator PBMC. As shown in Figure 1C, Treg populations isolated with the CliniMACS^8/25^ strategy were moderately suppressive, reaching 50% suppression at a 1∶1 Tresp∶Treg ratio, while MiniMACS isolated Treg populations were more potent, with 50% suppression at Tresp∶Treg ratios of approximately 4∶1.

### Expansion of CliniMACS Treg with either polyclonal or alloantigen stimulation increases cell numbers while suppressive activity is retained

CliniMACS Treg isolation strategies yield high numbers of Treg, but these numbers might still be too low for therapeutic purposes. This issue can be overcome by *ex vivo* expansion of Treg. To analyze expansion potential and effects of expansion on phenotypic and functional characteristics, CliniMACS^8/25^ isolated Treg populations were activated using either polyclonal stimulation (anti-CD3+anti-CD28 mAb coated microbeads) or alloantigen stimulation (irradiated PBMC from an HLA-mismatched donor), in the presence of exogenous IL-2.

**Figure 2:**
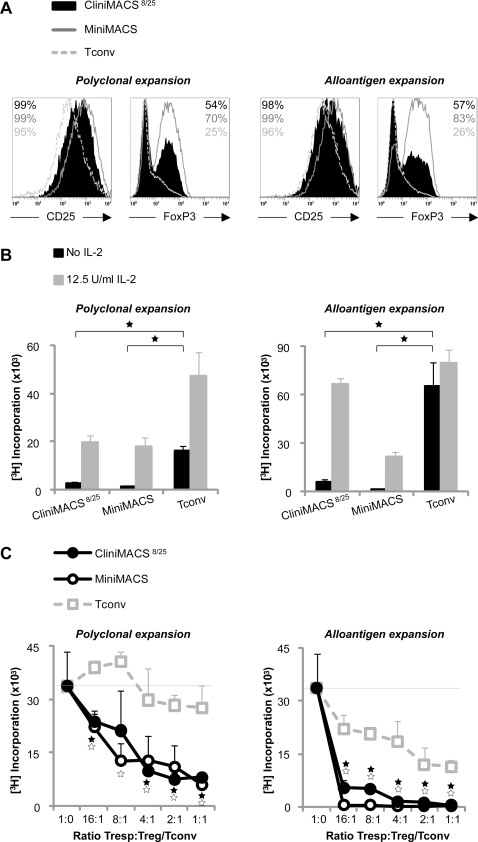
Phenotypic and functional characteristics of expanded CliniMACS isolated Treg. Cell populations were expanded with polyclonal stimulus or alloantigen stimulus in the presence of exogenous IL-2. Data from a typical experiment are shown (N = 3). (A) Cell surface expression of CD25 and intracellular expression of FoxP3. (B) Proliferative capacity upon restimulation with allogeneic PBMC (same donor as in alloantigen expansion) in the absence or presence of exogenous IL-2, measured at day 3 of culture. (C) Suppressive capacity in co-cultures of autologous naïve CD4^pos^CD25^neg^ Tresp stimulated with allogeneic PBMC (same donor as in alloantigen expansion). Significant differences are indicated by asterisks. In figure C, open and filled asterisks refer to Tconv (expanded with same stimulus as Treg populations) versus MiniMACS or CliniMACS^8/25^, respectively.

Polyclonal and alloantigen stimulated expansion cultures yielded similar cell numbers (Table 2). CliniMACS^8/25^ populations increased about 30-fold in cell numbers in ten days. The phenotype of expanded CliniMACS^8/25^ populations is depicted in Figure 2A. CD25 expression was high in both CliniMACS^8/25^ and MiniMACS populations, while the expression of FoxP3 was lower in expanded CliniMACS^8/25^ cells as compared to expanded MiniMACS populations. After expansion, all Treg populations remained anergic (Figure 2B). CliniMACS^8/25^ and MiniMACS populations suppressed alloantigen driven responder T cell proliferation to a similar extent (Figure 2C). Notably, alloantigen expanded Treg populations showed higher suppressive capacity in alloantigen driven responder T cell responses as compared to polyclonally expanded Treg (>75% suppression at a Tresp∶Treg ratio of 16∶1 versus 50% suppression at Tresp∶Treg ratios a Tresp∶Treg ratio of 8∶1, respectively, Figure 2C). This indicates that the alloantigen expanded cell populations were enriched for alloantigen-specific Treg. As our group has shown previously, the low proliferation observed after addition of alloantigen expanded Tconv can be explained by distinct culture kinetics with an earlier proliferation peak and a net result of lower counts at day 5 [21].

**Table 2:** Increase in CliniMACS Treg numbers by expansion.

	Polyclonal	Alloantigen
**CliniMACS^8/25^**	31 (16)	28 (14)
**CliniMACS^8/19/25^**	34 (17)	34 (11)
**MiniMACS**	4 (0)	9 (3)
**Tconv**	46 (1)	55 (10)

Fold increase in cell numbers (SD) after 10 days expansion with indicated stimulus in the presence of exogenous IL-2, N = 3.

**Figure 3:**
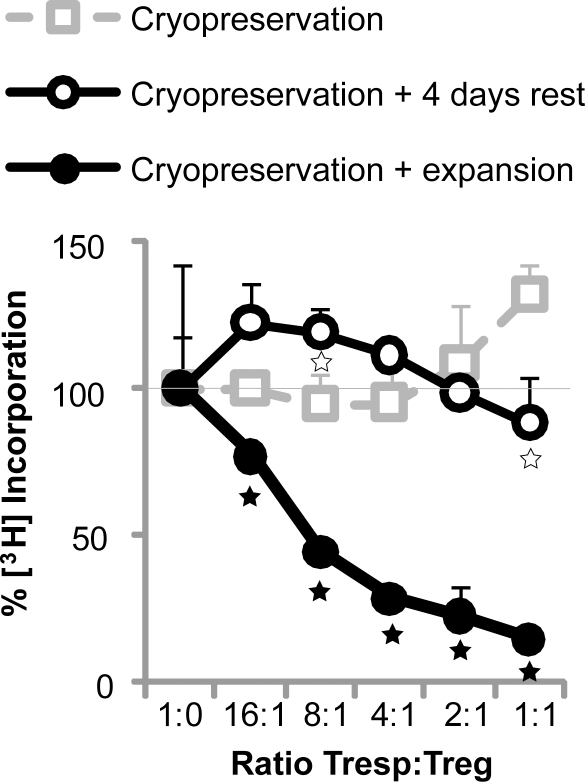
Cryopreservation of CliniMACS Treg affects suppressive capacity, which can be restored by activation. Suppressive capacity of cryopreserved CliniMACS Treg in co-cultures of autologous naïve CD4^pos^CD25^neg^ Tresp stimulated with allogeneic PBMC. Data from a typical experiment are shown (N = 2–4). Prior to co-culture suppression assay, indicated cell populations were expanded for 10 days in the presence of exogenous IL-2 and allogeneic PBMC (same donor as in co-culture suppression assay). Significant differences are indicated by asterisks. Open and filled asterisks refer to cryopreservation versus cryopreservation plus 4 days rest or cryopreservation plus expansion, respectively.

It would be convenient for Treg immunotherapy to be able to store Treg cells prior to manipulation and/or infusion. The suppressive capacity of cryopreserved CliniMACS Treg was impaired (Figure 3), and could not be regained by cell resting for up to seven days in culture medium. However, expansion of cryopreserved CliniMACS cells restored suppressive capacity.

### Depletion of CD19^pos^ cells during CliniMACS Treg isolation is necessary to prevent B cell contamination

**Figure 4:**
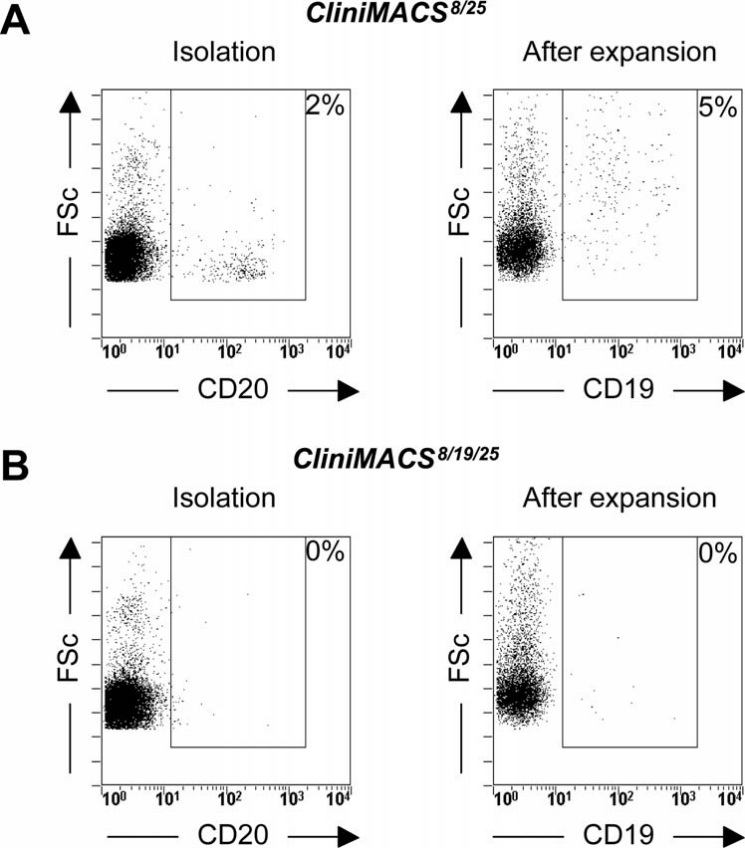
B cell contamination can be prevented by depleting CD19^pos^ cells during CliniMACS Treg isolation. Percentage of contaminating B cells in freshly isolated or alloantigen expanded CliniMACS populations. Data from a typical experiment are shown (N = 3). (A) CliniMACS^8/25^ populations. (B) CliniMACS^8/19/25^ populations.

After CliniMACS^8/25^ isolation procedures, less than 3% B cells were present in isolated populations, these numbers increased after expansion to 5 to 10% (Figure 4A). In order to remove B cells from our CliniMACS isolated Treg populations, we tested the efficacy of CD19^pos^ B cell depletion in the CliniMACS isolation procedure. In this strategy, first CD8^pos^ and CD19^pos^ cells were depleted, followed by enrichment of CD25^pos^ cells (referred to as CliniMACS^8/19/25^). Less than 0.5% B cells were present after these isolations (see Table 1 and Figure 4B) and no substantial B cell contaminations arose after either polyclonal or alloantigen expansion (see Table 2 and Figure 4B). CliniMACS^8/19/25^ populations were similar to CliniMACS^8/25^ cells with respect to the phenotype, anergic state and suppressive potential, both directly after isolation as well as after expansion (data not shown). Thus, CD19^pos^ depletion effectively prevents B cell contaminations in CliniMACS Treg populations after isolation and after expansion.

### Depletion of CD127^pos^ cells improves CliniMACS Treg purity

**Figure 5:**
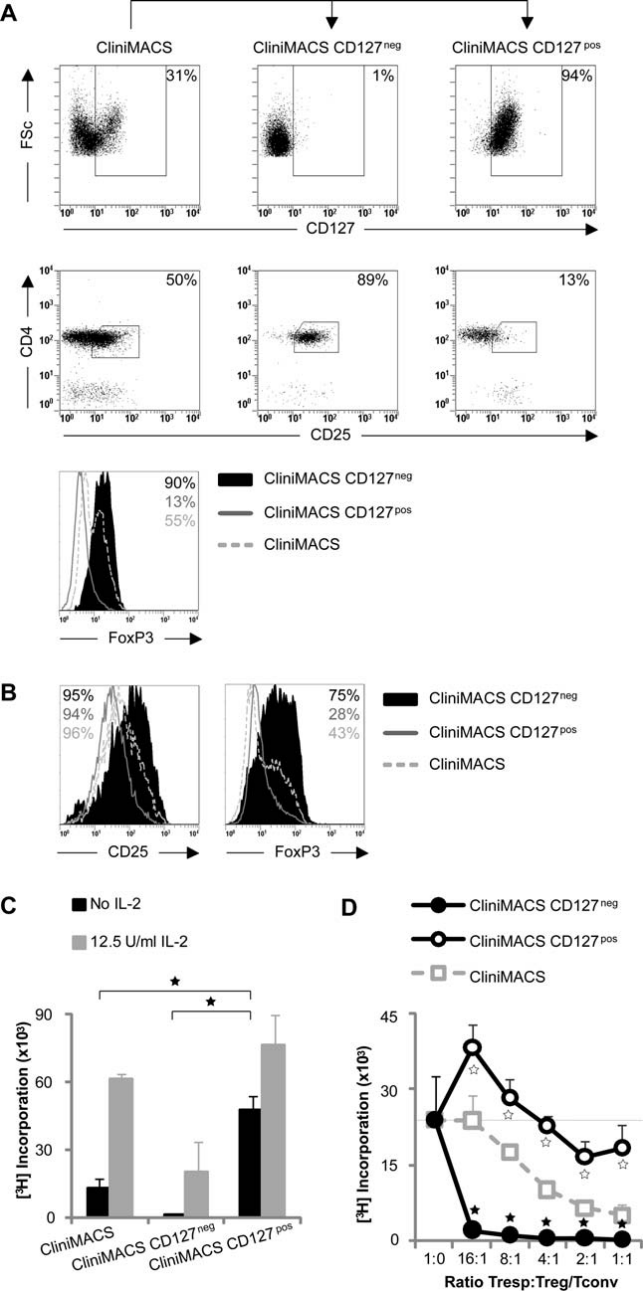
CliniMACS Treg isolation can be improved by depleting CD127^pos^ Tconv cells. CliniMACS populations were stained with anti-CD127 and sorted into CD127^neg^ and CD127^pos^ subsets. CliniMACS cells were cryopreserved prior to cell sorting. Data from one of two similar experiments are shown. (A) Cell surface expression of CD127, CD4 and CD25 and intracellular expression of FoxP3. (B-C-D) CliniMACS populations were expanded with alloantigen stimulation and exogenous IL-2. (B) Cell surface expression of CD25 and intracellular expression of FoxP3. (C) Proliferative capacity upon restimulation with allogeneic PBMC (same donor as in alloantigen expansion) in the absence or presence of exogenous IL-2. (D) Suppressive capacity in co-cultures of autologous naïve CD4^pos^CD25^neg^ Tresp stimulated with allogeneic PBMC (same donor as in alloantigen expansion). Significant differences are indicated by asterisks. In figure D, open and filled asterisks refer to CliniMACS versus CliniMACS CD127^pos^ or CliniMACS CD127^neg^, respectively.

CD4^pos^CD25^neg/low^FoxP3^neg/low^ Tconv cells were the major contaminating cell type in CliniMACS isolated Treg (40–60% of the isolated populations, see Figure 1). Recently, it has been described that Tconv express the IL-7 receptor alpha-chain CD127, while Treg cells do not express CD127 [22], [23]. Indeed, CliniMACS isolated populations contained both CD127^neg^ and CD127^pos^ cells, reflecting the presence of both Treg and Tconv cells (Figure 5A). To improve the purity of Treg isolated with CliniMACS, we hypothesized that depletion of CD127^pos^ cells could be employed to exclude contaminating T cells. Because anti-CD127 CliniMACS beads are currently not available, we depleted CD127^pos^ cells from CliniMACS isolated cell populations by FACS.

As shown in Figure 5A, this procedure indeed enabled separation of Treg from Tconv cells. In contrast to CD127^pos^ cells, the majority of CliniMACS CD127^neg^ cells showed a Treg phenotype: CD4^pos^CD25^high^ and FoxP3^pos^. Since we used cryopreserved CliniMACS populations for these experiments, we expanded the populations prior to functional characterization. After expansion, CD127^neg^ populations retained a high Treg purity, as evidenced by high expression of CD25 and FoxP3 (Figure 5B). In contrast to CD127^pos^ populations, CD127^neg^ populations were anergic after expansion (Figure 5C). As expected, CD127 depletion enhanced suppressive potential, as shown by the significantly higher suppressive capacity of CD127^neg^ populations as compared to CliniMACS populations that were not depleted of CD127^pos^ cells (Figure 5D).

## Discussion

Immunotherapy using *ex vivo* activated and/or expanded CD4^pos^CD25^high^ regulatory T cells appears promising, not only in stem cell transplantation, but also for a number of autoimmune disorders. To be optimally effective, the availability of tailor-made clinical grade procedures is a prerequisite. As previously reported [14], [15], CliniMACS GMP Treg isolation strategies based on CD25^pos^ enrichment typically result in 40–60% pure Treg with moderate suppressive activity.

The degree of purity of Treg that is required may depend on the actual application. In stem cell transplantation, the first clinical trials on Treg immunotherapy have recently been initiated. In these studies, either CliniMACS isolated CD4^pos^CD25^high^ Treg (with 40–60% purity) or *ex vivo* manipulated CD4^pos^ T cell lines consisting of 5–10% induced regulatory Tr1 cells and consequently a substantial amount of non-Treg were infused in patients receiving stem cell transplantations [24]. So far, to our knowledge, no adverse effects have been reported. However, caution is necessary when applying Treg for auto-immune disease or solid organ transplant purposes. Here an inadvertently present effector pool might cause serious pathology.

As cytotoxic CD8^pos^ T cells are a potentially aggressive cell type in immune responses, it is highly recommendable to exclude these cells from CliniMACS isolated Treg populations. Here, we show that the CD8^pos^ CliniMACS cell depletion step was effective: less than 0.1% CD8^pos^ cells were present in isolated Treg populations, and no outgrowth of CD8^pos^ cells occurred after expansion of CliniMACS Treg populations. Treg isolated by CD25^pos^ enrichment following CD8^pos^ depletion contained a small contamination of B cells (1–3%), which could increase to up to 10% after T cell expansion. The risks of infusion of expanded and activated autologous B cells are unknown, but could in theory include the development of a B cell lymphoproliferative disorder that can occur after transplantation and is often lethal [25]–[27]. We were able to prevent the contamination by B cells by including a CD19^pos^ B cell depletion step in the isolation strategy.

Notwithstanding the favorable effects of combined anti-CD25, anti-CD8 and anti-CD19 microbead isolation (the currently available clinical grade tools), the isolated Treg population was of suboptimal purity. Most of the contaminating cells appeared to be CD4^pos^CD25^neg/low^ Tconv cells that could potentially lead to immunopathology. To further improve CliniMACS Treg purity, Wichlan *et al.* studied titration of anti-CD25 microbeads in the enrichment phase of CliniMACS Treg isolation. While the use of lower amounts of beads indeed led to populations with higher CD25 expression levels [15], the suppressive capacity of the isolated populations did not improve and cell yields were significantly lower, indicating that titration of CD25 microbeads will not provide the solution to achieve higher purity for CliniMACS Treg isolation. These authors also reported that adjustment of CliniMACS flow rates did not lead to a higher Treg purity. Hoffmann *et al.* used three consecutive CliniMACS selection cycles for CD25^pos^ cells, to preferentially select CD25^high^ Treg and exclude CD25^neg/low^ Tconv [14]. However, the degree of Treg purity obtained with this approach was similar to that achieved with one CD25 enrichment cycle.

Recently, it has been described that Tconv express the IL-7 receptor alpha chain CD127, while Treg do not express this molecule [22], [23]. Thus, in the current study, we hypothesized that the conventional T cells contaminating GMP Treg populations could be excluded from the CliniMACS isolated populations by depleting CD127^pos^ cells. Since anti-CD127-microbeads for use in CliniMACS are currently not available, we depleted CD127^pos^ cells from CliniMACS isolated Treg populations by FACS. This procedure indeed increased Treg purity, as shown by uniformly high expression of FoxP3 in CD127^neg^ populations. After expansion of CliniMACS CD127^neg^ populations, high expression of FoxP3^pos^ was maintained, and the cell populations showed potent suppressive capacity. Notably, suppressive capacity of CliniMACS CD127^neg^ populations was significantly higher than that of non-depleted CliniMACS populations, indicating that CD127 depletion clearly enriched for functional Treg.

The outcome of a particular T cell based immune response is likely to be determined by the balance between the effector and regulatory T cell pools. Consequently, the efficacy of Treg therapy may be determined by the actual number of antigen reactive Treg within the infused cell population. Indeed, antigen-specific Treg were proven to be more efficient than polyclonal Treg in preclinical mouse models of autoimmunity [6], [12], [13] and graft-versus-host disease [3], [5], [7]. Previously, we and others have shown that human Treg isolated by non-clinical grade strategies can be expanded using both polyclonal and alloantigen stimulation methods [16]–[21]. Importantly, we show in this report that this also holds true for clinical grade isolated Treg. Due to the moderate purity of CliniMACS isolated Treg populations, outgrowth of contaminating cells could be a risk upon expansion. Indeed, expanded CliniMACS^8/19/25^ Treg populations contained a significant percentage of FoxP3^neg^ cells, however, this could be prevented by depletion of CD127^pos^ cells from the CliniMACS isolated Treg populations.

Clinical implementation of Treg based therapy will be highly facilitated if Treg can be stored prior to infusion, as this will allow a more flexible timing of Treg therapy and/or therapeutic schemes with multiple Treg treatments over time. We studied the feasibility of CliniMACS Treg cryopreservation in liquid nitrogen. Results indicate that Treg can survive cryopreservation, as thawed populations showed 70–80% cell viability. We noted decreased suppressive activity of thawed Treg populations, which could be restored by Treg expansion. An alternative approach would be to expand Treg prior to cryopreservation. This resulted in unaltered suppressive capacity upon thawing, at least in MiniMACS isolated Treg (Supplemental data, Figure S1).

In summary, we here provide further support for clinical implementation of Treg immunotherapy by showing that a high Treg purity can be reached, and that isolated cells can be cryopreserved and expanded successfully.

## Materials and Methods

### Human cells

Healthy donors were scheduled for leukapheresis procedures to obtain leukocytes for donor lymphocyte infusions in hematopoietic stem cell transplantation patients in the Radboud University Nijmegen Medical Centre. Excess leukapheresis material was used for the current study upon written informed consent with regard to scientific use. Buffy coats from healthy human donors were purchased from Sanquin bloodbank, Nijmegen, The Netherlands, upon written informed consent with regard to scientific use and used as a source of stimulator PBMC. The current study did not require approval from an ethics committee according to the Dutch Medical Research Involving Human Subjects Act.

### Treg isolation

Healthy donor leukapheresis products were used for CliniMACS CD4^pos^CD25^high^ Treg isolation. Cells were washed with PBS/EDTA buffer (Miltenyi Biotec, Bergisch-Gladbach, Germany, supplemented with 0.5% HSA (Sanquin bloodbank). Anti-CD8 and/or anti-CD19 coated CliniMACS microbeads (kindly provided by Miltenyi Biotec) were added, incubated for 30 minutes and washed. CliniMACS program 2.1 was run to deplete labeled cells (CliniMACS separation columns were kindly provided by Miltenyi Biotec). The labeling procedure was repeated with anti-CD25 CliniMACS microbeads, and CliniMACS program 1.1 was run to enrich for CD25^pos^ cells.

A non-GMP MiniMACS based CD4^pos^CD25^high^ Treg isolation method was performed on leukocytes from the same leukapheresis products as used for CliniMACS Treg isolation. PBMC were isolated by density gradient centrifugation (Lymphoprep, Nycomed Pharma, Roskilde, Denmark). CD4^pos^ T cells were negatively selected using mAbs directed against CD8 (RPA-T8), CD14 (M5E2), CD16 (3G8), CD19 (4G7), CD33 (P67.6), CD235a (GA-R2(HIR2) (BD Biosciences, San Jose, CA, USA), and CD56 (MOC-1) (Dako, Glostrup, Denmark) combined with sheep-anti-mouse-IgG coated magnetic beads (Dynal Biotech, Oslo, Norway), routinely resulting in a >90% pure CD4^pos^ T cell fraction. CD25^high^ Treg and CD25^neg^ conventional T cells (referred to as Tconv, included in all experiments as control cell population) were separated by MACS-sorting, using 10 µl anti-CD25 magnetic microbeads/10^7^ CD4^pos^ cells (Miltenyi Biotec).

For CD127^pos^ cell depletion, CliniMACS isolated Treg were stained with anti-CD127-AlexaFluor647 (BD Biosciences) and CD127^pos^ cells were depleted on an Elite FACS machine (Beckman Coulter, Fullerton, CA, USA).

Stimulator PBMC were isolated from healthy donor buffy coat by density gradient centrifugation (Lymphoprep, Nycomed Pharma).

HLA typing was performed by serological and DNA based techniques according to international (ASHI/EFI) standards [28].

### Treg cryopreservation

Treg cells were suspended in RPMI 1640 supplemented with pyruvate (0,02 mM), penicillin (100 U/ml), streptomycin (100 mg/ml), 20% human pooled serum (HPS) and 15% dimethylsulfoxide, were kept in −80°C for one week, and were subsequently transferred to liquid nitrogen for up to one year. Cells were quickly thawed in a 37°C water bath and washed twice before use.

### Treg expansion

Cell cultures were performed in culture medium consisting of RPMI 1640 supplemented with pyruvate (0.02 mM), penicillin (100 U/ml), streptomycin (100 mg/ml), 10% HPS and IL-2 (25 U/ml, Chiron, Amsterdam, the Netherlands) with 5×10^4^ cells per well in 96-well round bottom plates, in a 37°C, 95% humidity, 5% CO_2_ incubator. Stimulation was provided by either 10^5^ irradiated (30 Gy) fully HLA-mismatched allogeneic PBMC per well (alloantigen expansion) or 1×10^4^ anti-CD3+anti-CD28 mAb coated microbeads per well (Dynal Biotech, polyclonal expansion).

Wells were split and provided with fresh medium containing cytokines every 3 days. After 10 days, the cells were harvested, washed and rested for 2 days in 5% HPS culture medium before functional and phenotypic analyses.

### Flow cytometry

The phenotype of cells was analyzed by five-color flow cytometry (FC500, Beckman Coulter). For cell surface staining, the following conjugated mAbs were used: anti-CD4(SFCI12T4D11)-PCy7, anti-CD19(J4.119)-PE (Beckman Coulter), anti-CD25(4E3)-biotin, anti-biotin(Bio3-18E7)-PE (used in Figures 1 and 2), anti-CD20(LT20)-PE (Miltenyi Biotec), anti-CD25(M-A251)-PE (used in Figure 5), and anti-CD127(hIL-7R-M21)-AlexaFluor647 (BD Biosciences). For intracellular staining, Fix and Fix/Perm buffer and anti-FoxP3(PCH101)-FITC were used according to the manufacturer's instructions (eBioscience, San Diego, CA, USA). Isotype controls were used for gate settings.

### Stimulation assay to analyze T cell anergy

T cell anergy was examined in (re-)stimulation assays. 2.5×10^4^ cells were stimulated with 10^5^ irradiated allogeneic stimulator PBMC in the presence or absence of IL-2 (12.5 U/ml). Proliferation was measured at day 5 (primary responses) or day 3 (secondary responses). To this end, 0.5 µCi [^3^H]Thymidine (Amersham Biosciences, Piscataway, NJ) was added to each well. After 8 hours, [^3^H]Thymidine incorporation was measured using a beta-plate counter (Packard, Canberra, Australia). Tests were set up in triplicate; results were expressed as mean+SD counts per 5 minutes.

### Co-culture suppression assays

Suppressive capacity of Treg was studied in co-culture suppression assays. 5×10^4^ autologous responder T cells (CD4^pos^CD25^neg^, Tresp) were stimulated with 10^5^ irradiated allogeneic stimulator PBMC. Treg were titrated into these cultures. Proliferation was measured at day 5 by determination of [^3^H]Thymidine incorporation as described above. Tests were set up in triplicate, results were expressed as mean+SD counts per 5 minutes.

### Statistical analysis

Proliferative capacities of different cell populations were compared using Student T tests. A P value<0.05 was considered statistically significant.

## Supporting Information

Figure S1 **Unaffected suppressive capacity of MiniMACS Treg by expansion prior to cryopreservation.** Suppressive capacity of cryopreserved MiniMACS Treg isolated from healthy donor buffy coat in co-cultures of autologous naïve CD4^pos^CD25^neg^ Tresp stimulated with allogeneic PBMC. Data from one of two similar experiments are shown. Prior to co-culture suppression assay, indicated cell populations were expanded for 10 days in the presence of exogenous IL-2 and allogeneic PBMC (same donor as in co-culture suppression assay). Significant differences are indicated by asterisks. Open and filled asterisks refer to cryopreservation versus cryopreservation followed by expansion or expansion followed by cryopreservation, respectively.
(0.12 MB TIF)
