# Supplementary figures and images for: Clinical Grade Treg: GMP Isolation, Improvement of Purity by CD127pos Depletion, Treg Expansion, and Treg Cryopreservation
Source: PLoS One. 2008 Sep 8;3(9):e3161. doi: 10.1371/journal.pone.0003161 (PMC2522271; doi:10.1371/journal.pone.0003161)

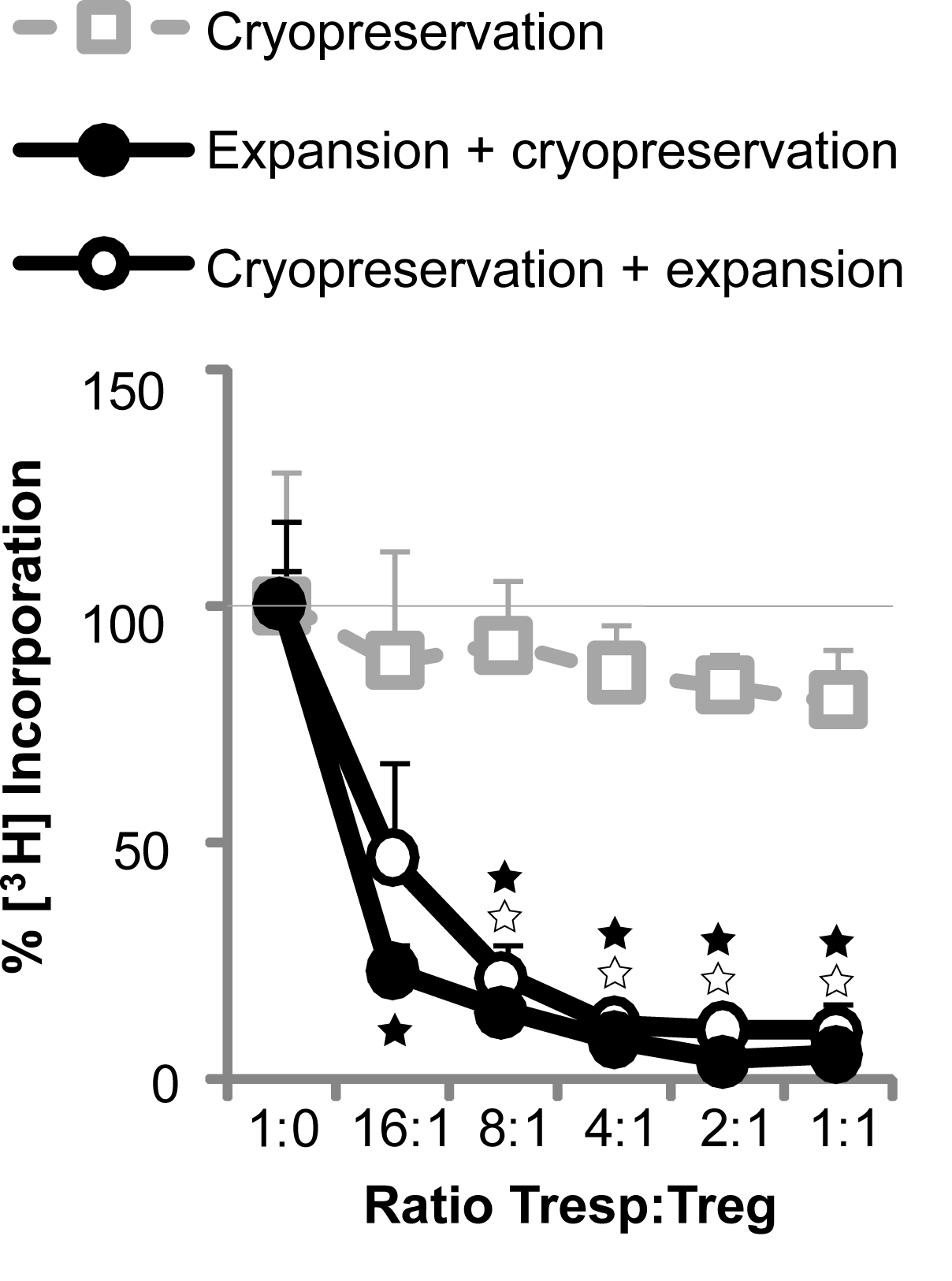

Supplement: Figure S1 — Unaffected suppressive capacity of MiniMACS Treg by expansion prior to cryopreservation. Suppressive capacity of cryopreserved MiniMACS Treg isolated from healthy donor buffy coat in co-cultures of autologous naïve CD4posCD25neg Tresp stimulated with allogeneic PBMC. Data from one of two similar experiments are shown. Prior to co-culture suppression assay, indicated cell populations were expanded for 10 days in the presence of exogenous IL-2 and allogeneic PBMC (same donor as in co-culture suppression assay). Significant differences are indicated by asterisks. Open and filled asterisks refer to cryopreservation versus cryopreservation followed by expansion or expansion followed by cryopreservation, respectively. (0.12 MB TIF) [file pone.0003161.s001.tif]
